# Particle Size Concentration Distribution and Influences on Exhaled Breath Particles in Mechanically Ventilated Patients

**DOI:** 10.1371/journal.pone.0087088

**Published:** 2014-01-27

**Authors:** Gwo-Hwa Wan, Chieh-Liang Wu, Yi-Fang Chen, Sheng-Hsiu Huang, Yu-Ling Wang, Chun-Wan Chen

**Affiliations:** 1 Department of Respiratory Therapy, College of Medicine, Chang Gung University, Tao-Yuan, Taiwan; 2 Department of Internal Medicine, Taichung Veterans General Hospital, Chiayi Branch, Chiayi, Taiwan; 3 Department of Respiratory Therapy, Chung Shan Medical University Hospital, Taichung, Taiwan; 4 Institute of Occupational Medicine and Industrial Hygiene, College of Public Health, National Taiwan University, Taipei, Taiwan; 5 Department of Respiratory Therapy, Taichung Veterans General Hospital, Taichung, Taiwan; 6 Instititute of Occupational Safety and Health, Council of Labor Affairs, New Taipei City, Taiwan; Alberta Provincial Laboratory for Public Health/University of Alberta, Canada

## Abstract

Humans produce exhaled breath particles (EBPs) during various breath activities, such as normal breathing, coughing, talking, and sneezing. Airborne transmission risk exists when EBPs have attached pathogens. Until recently, few investigations had evaluated the size and concentration distributions of EBPs from mechanically ventilated patients with different ventilation mode settings. This study thus broke new ground by not only evaluating the size concentration distributions of EBPs in mechanically ventilated patients, but also investigating the relationship between EBP level and positive expiratory end airway pressure (PEEP), tidal volume, and pneumonia. This investigation recruited mechanically ventilated patients, with and without pneumonia, aged 20 years old and above, from the respiratory intensive care unit of a medical center. Concentration distributions of EBPs from mechanically ventilated patients were analyzed with an optical particle analyzer. This study finds that EBP concentrations from mechanically ventilated patients during normal breathing were in the range 0.47–2,554.04 particles/breath (0.001–4.644 particles/mL). EBP concentrations did not differ significantly between the volume control and pressure control modes of the ventilation settings in the mechanically ventilated patients. The patient EBPs were sized below 5 µm, and 80% of them ranged from 0.3 to 1.0 µm. The EBPs concentrations in patients with high PEEP (> 5 cmH_2_O) clearly exceeded those in patients with low PEEP (≤ 5 cmH_2_O). Additionally, a significant negative association existed between pneumonia duration and EBPs concentration. However, tidal volume was not related to EBPs concentration.

## Introduction

Human lungs emit non-volatile aerosols and volatile organic compounds in exhaled breath. Humans produce exhaled breath particles (EBPs) according to their respiratory patterns. The mechanisms of EBPs production include: production of wind shear forces in the respiratory system under high speed flow conditions, like coughing, sneezing, singing, laughing, and talking, with EBPs subsequently exiting via the attached airway mucosa [1], [2]; airflow passes through the oropharyngeal bifurcation during normal breathing and then becomes turbulent flow that produces aerosols [3]; bronchiole fluid film burst (BFFB) results from the reopening of terminal airways following deep exhalation, with airflow then passing through the film and causing EBPs to break out [3], [4].

Several studies evaluated the characterization of concentration and size distribution of EBPs [5], [6]. Exhaled breath particles concentrations were associated with human activity patterns and varied among individulas. EBPs concentration was positively associated with tidal volume, ventilation ratio [tidal volume (V_T_)/vital capacity (VC)] [2], deep exhalation [4], and long breath holding at residual volume [7]. However, the ratio of functional residual capacity to total lung capacity (> 0.45) [2] and short breath holding at total lung capacity [7] were negatively associated with the EBPs concentrations. Additionally, inspiratory and expiratory flow did not affect the emission rate of EBPs [2]. Previous studies indicated that the maximum and minimum concentrations of EBPs during normal breathing in healthy individuals were 0.15–1,383 droplets/mL [5], [6], [8]–[10] and 0.01–2.1 droplets/mL [1], [5], [6], respectively. Additionally, previous studies focused on healthy adults found that the highest EBPs concentrations occurred during sneezing [11] and coughing [6], [11] while the lowset occurred during nasal breathing [6] and talking [11].

Exhaled particle size distribution was in the interval 0.3–2.0 µm [4]. A previous study showed that the count median diameter of EBPs was 0.28 µm, and only 2% of EBPs were larger than 0.5 µm [2]. The mean particle sizes of EBPs were less than 1 µm during normal breathing [1], [5], [6] and 1–125 µm during coughing [5], [6], [8]–[10]. Particle size is a key factor for disease transmission [12]. Patients with airway infection may exhale particles with pathogens, thus increasing airborne transmission risk. A previous study showed that 25% of patients with pulmonary tuberculosis exhaled 3–633 CFU (colony forming unit) of *Mycobacterium tuberculosis* when coughing, and levels of this pathogen primarily ranged 0.6–3.3 µm. The level of exhaled particles with *Mycobacterium tuberculosis* during coughing clearly decreased following one week of treatment with anti-truberculosis drugs [13].

In clinical practice, patients with acute respiratory failure or severe diseases (chronic obstructive pulmonary disease, neuromuscular disease, etc.) must use ventilators for life support [14], [15]. Ventilator mode was set based on patient clinical condition, operator familiarity, and hopital preference. The pressure control (PC) mode and volume control (VC) mode settings are commonly used in hospital ventilator systems. Patient inspiratory flow and volume are changed by in response to patient ventilation demand under pressure control mode setting, while under the volume control mode setting patients are provided with fixed inspiratory flow and volume.

To date, most studies focused on EBPs concentration distributions in healthy individuals with various respiratory patterns and performing various activities. Few studies assessed the relationships among patient characteristics, ventilator mode setting, and size concentration distributions of EBPs. Therefore, this investigation focused on the relationships among size concentration distribution of EBPs, ventilator setting (mode, tidal volume, and PEEP), and pulmonary disease in mechanically ventilated patients.

## Materials and Methods

### Study population

Mechanically ventilated patients aged over 20 years were recruited from the respiratory intensive care unit of a medical center in central Taiwan. These patients used pressure control mode (n = 24) or volume control mode (n = 24) for ventilation control. This investigation was approved by the Institutional Review Board of Taichung Veterans General Hospital. Informed written consent was obtained from the families of each subject before their participation.

### Measurement of exhaled breath particles

**Figure 1:**
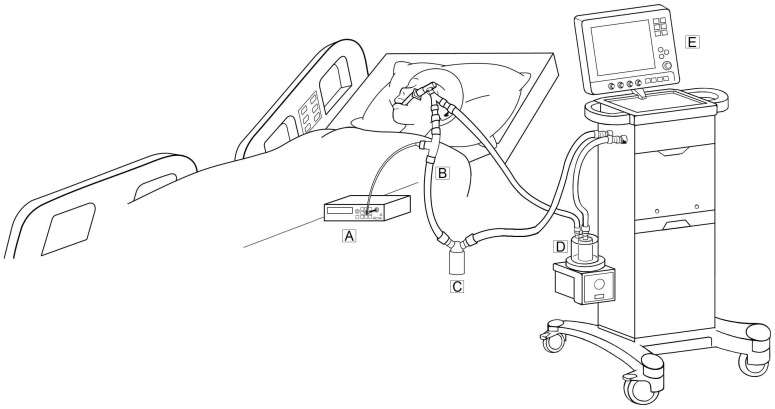
Diagram of exhaled breath particles sampling in mechanically ventilated patients. A: portable DUSTcheck monitor, B: expiratory breathing circuit, C: water trap, D: heated humidifier, E: Mechanical ventilator system.

To ensure accurate measurement of EBPs concentrations, this study used a lung model to assess the background particle concentrations of the ventilator circuit system using the central piping air system. The PB840 (Puritan Bennett 840, Tyco Healthcare, Mansfield, MA) and Servo-i (Maquet, Inc., Bridgewater, NJ) ventilators were used for the lung model testing and identical ventilation parameters were set. For the volume control mode, the ventilation parameter settings in the lung model included tidal volume of 500 mL, respiratory rate of 14 breaths/minute, peak inspiratory flow of 50 liters/minute, and inhaled oxygen concentration of 40%. The Servo-i ventilator has an automatic control system and so there was no fixed inspiratory flow. The tidal volume and ventilation parameter levels were observed in evaluation of the leakage of the ventilator circuit systems. A portable DUSTcheck monitor (Model 1.108; Grimm Labortechnik Ltd., Ainring, Germany) was used to measure the particle concentrations in the ventilator circuit systems. The DUSTcheck monitor detects aerosol particles in the size range of 0.3–20 µm in 15 size channels and represents the results in number concentration. The sample flow rate of the DUSTcheck monitor was 1.2 L/min which was only higher than the air flow rate in the first and the last 0.1 second during an exhalation maneuver. The particle concentrations were recored every 6 seconds. The air sampling for each test lasted 7 minutes, and the mean value of 5-minute data was presented. The mean exhaled paritcle number concentrations were represented as number concentration (particles/mL) and multiplied by tidal volume to determine the total paricle amounts per breath (particles/breath) in each sample. Figure 1 shows the diagram of EBP sampling in the mechanically ventilated patients. In order to take representative samples independent of particle size, it is necessary to remove the sample stream isokinetically. Unfortunately, the velocity of air during expiratory activities varies widely with breathing pattern and time. This makes the isokinetic sampling unfeasible in this study.

### Clinical data collection

This study collected patient demographic and medical details (such as age, gender, height, weight, diagnosis, etc.) and record ventilation parameters (such as ventilator mode, inhaled oxygen concentration, tidal volume, inspiratory flow, PEEP, etc.) to assess the relationships among personal characteristics, ventilator settings, and EBPs concentrations in mechanically ventilated patients.

### Statistical analysis

Statistical analyses used SPSS version 13.0 (SPSS, Inc., Chicago, IL, USA). Figures were graphed using SigmaPlot 12.0 software (Systat software, Inc., San Jose, CA, USA) and GraphPad Prism 5.0 software (GraphPad Software, Inc., San Diego, CA, USA). The significance level for all tests was set to 0.05. The concentrations of EBPs were calculated based on 5-minute averages. Furthermore, the figures for PEEP level were assigned to a high level group (>5 cmH_2_O) and a low level group (≤5 cmH_2_O) using the median as the cutoff. The figures for tidal volume were also classified into high (>500 mL) and low (≤500 mL) level groups using the median as the cutoff. The nonparametric Kruskal-Wallis and Mann-Whitney U tests for non-normally distributed data were used to identify group differences in continuous variables.

## Results

This study measured compressed air from the central piping air system and found it to contain 0.001 particles/mL and 0.0023 particles/mL of particles sized above 0.3 µm for the PB840 and Servo-i ventilator systems, respectively. No particles were found in the piping air of the inspiratory breathing circuit system when a high efficiency particulate air (HEPA) filter was fitted to the ventilator system. Each experiment thus required a HEPA filter mounted in the inspiratory circuit of ventilator system.

The mechanically ventilated patients (24 with PC mode and 24 with VC mode) were aged between 52 and 91 years old. The patient heights and weights were 146–175 centimeters and 35–90 kilograms, respectively. Disease diagnoses inlcuded asthma, chronic obstructive pulmonary disease (COPD), encephalomyelitis, empyema, epilepsy, hepatoma, lung cancer, pneumonia, pulmonary tuberculosis (TB), rectal cancer, sepsis, septic shock, thoracolumbar compression fracture, and urosepsis (Table 1). The fractional inhaled oxygen concentration for mechanically ventilated patients was 30%–50%, and the setting of inhaled tidal volume depended on personal ideal body weight to achieve the target of 10 mL/kg. The patient respiratory rate ranged from 12 to 31 beats/minute, and the PEEP levels were 5–10 cmH_2_O in the mechanically ventilated patients. The mean ventilator-days in the mechanically ventilated patients with the PC and VC modes were 8.33 and 9.58 days, respectively.

**Table 1:** Personal characteristics and ventilator settings in mechanically ventilated patients with PC or VC modes.

Variables	PC mode	VC mode
Personal characteristics		
Sample size, n	24	24
Age, year	76.7 (2.15)	75.25 (2.50)
Sex, M/F	15/9	15/9
Height, cm	159.63 (1.85)	160.08 (1.69)
Weight, kg	57.87 (2.37)	57.41 (2.45)
Diagnosis		
Asthma	1	-
COPD	4	4
Encephalomyelitis	1	1
Empyema	1	-
Epilepsy	1	1
Hepatoma	1	2
Lung cancer	3	4
Pneumonia	9	12
Pulmonary TB	-	1
Rectal cancer	1	-
Sepsis	2	1
Septic shock	1	-
Thoracolumbar compression fracture	1	1
Urosepsis	1	2
Ventilator settings		
F_i_O2, %	30–40	30–50
Tidal volume, mL	479.52 (16.60)	490 (12.53)
Respiratory rate, breath/min	18 (0.51)	18 (0.49)
PEEP, cmH_2_O	5–10	5–10
Duration of ventilation, day	8.33 (1.10)	9.58 (2.17)

Data are presented with n or mean(sem). PC: pressure control; VC: volume control; COPD: chronic obstructive pulmonary disease; FiO_2_: fractional inhaled oxygen concentration; PEEP: positive expiratory end airway pressure.

**Figure 2:**
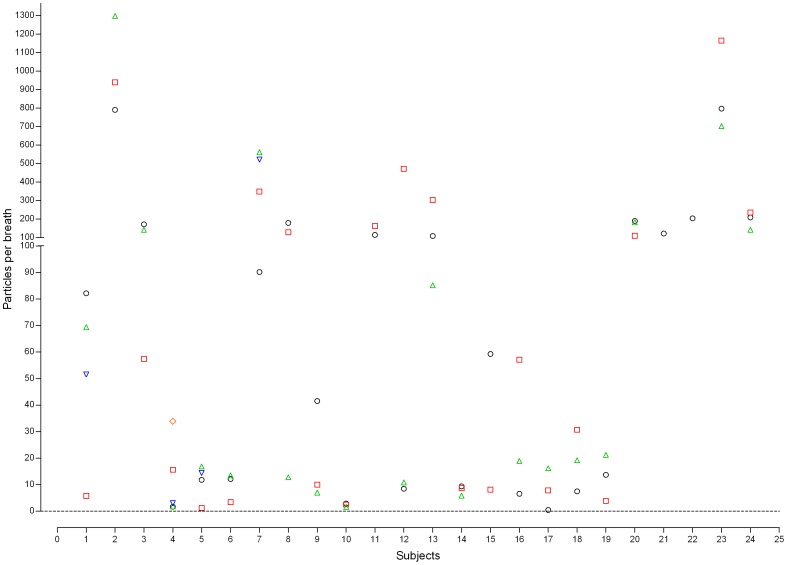
Exhaled breath particle concentration distributions in mechanically ventilated patients with the PC mode. PC: pressure control.

**Figure 3:**
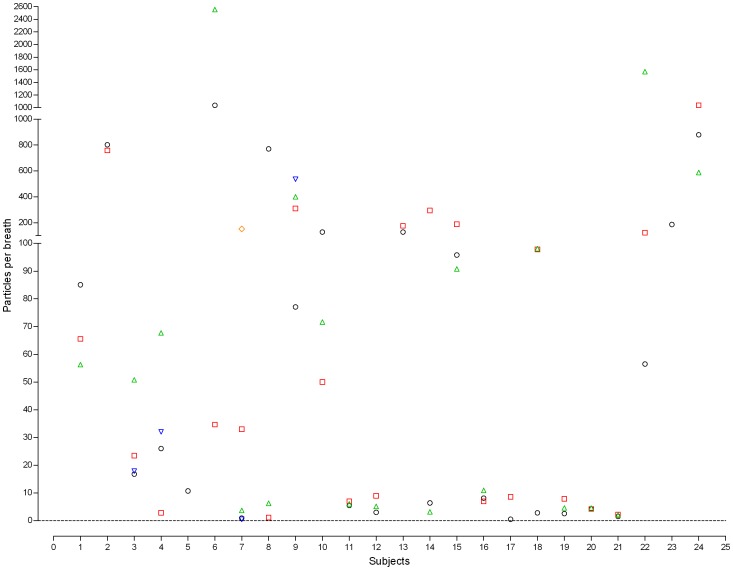
Exhaled breath particle concentration distributions in mechanically ventilated patients with the VC mode. VC: volume control.

**Figure 4:**
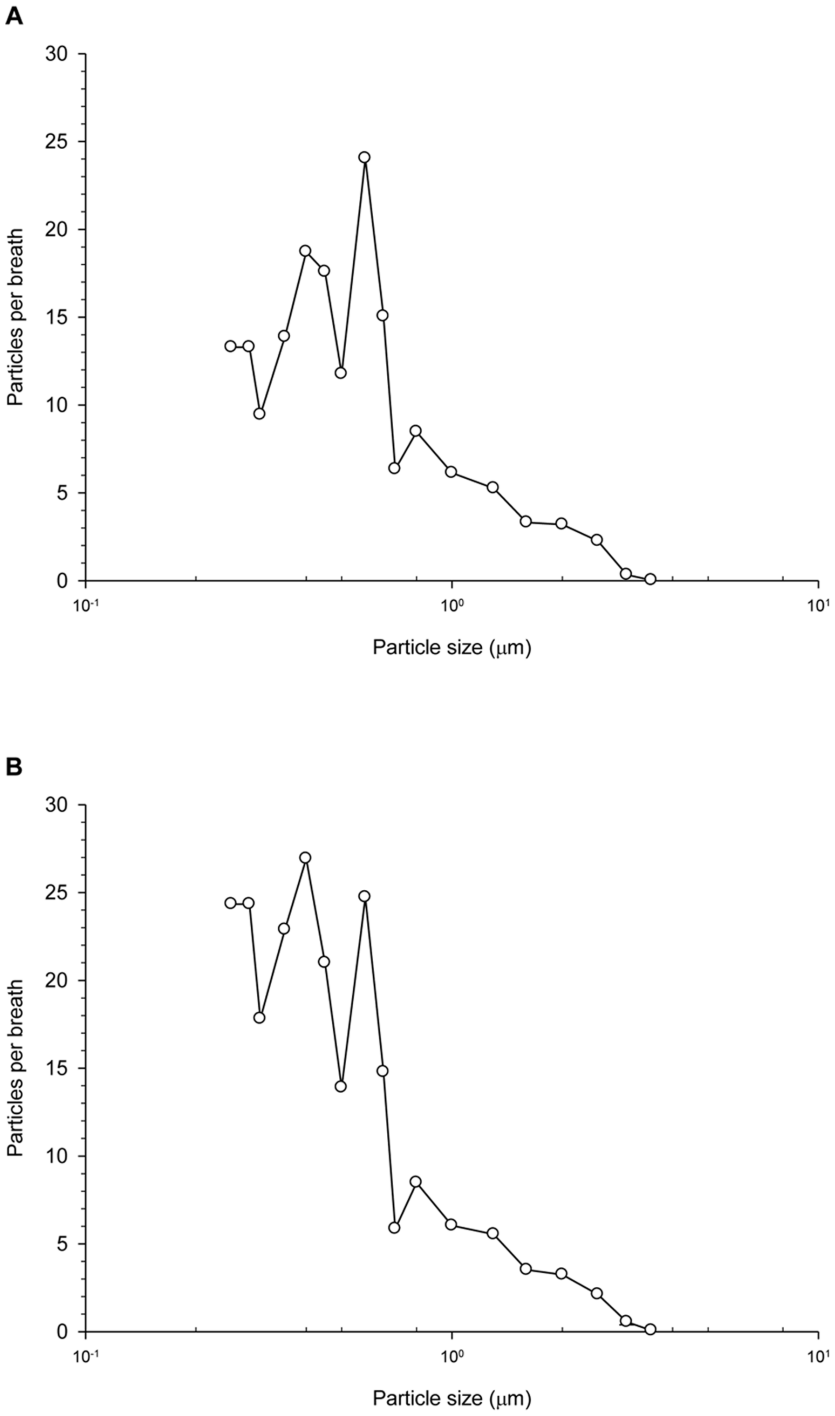
EBP size concentration distributions in mechanically ventilated patients with the PC or VC modes. (A) PC (pressure control) mode, (B) VC (volume control) mode.

This study found high variability of EBP concentrations among patients. The EBP concentrations for mechanically ventilated patients with PC mode during normal breathing were 0.47–1,163.87 particles/breath (0.001–2.527 particles/mL) (Fig. 2). Additionally, the EBP concentrations during normal breathing in the mechanically ventilated patients with the VC mode were 0.55–2554.04 particles/breath (0.001–4.644 particles/mL) (Fig. 3). The particle size concentration distributions from exhaled breath in the mechanically ventilated patients with the PC mode (Fig. 4A) resembled those of patients with the VC mode (Fig. 4B). Regardless of ventilator mode, most EBP from mechanically ventilated patients were smaller than 5 µm, and the highest and lowest EBPs concentrations occurred the size ranges 0.3–1.0 µm and 2.5–5.0 µm, respectively (data not shown).

**Figure 5:**
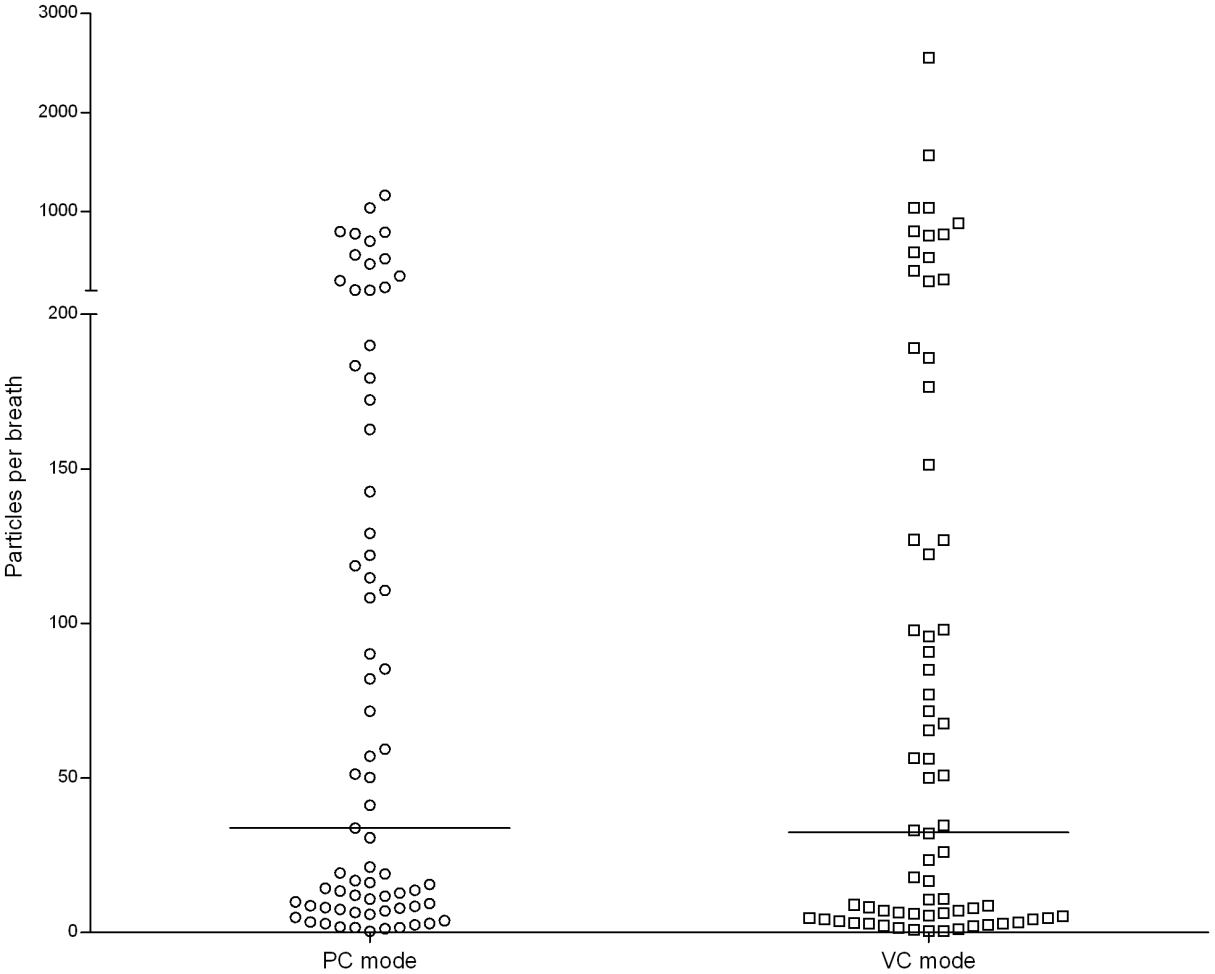
Comparison of EBP concentrations in patients with different ventilation modes.

**Figure 6:**
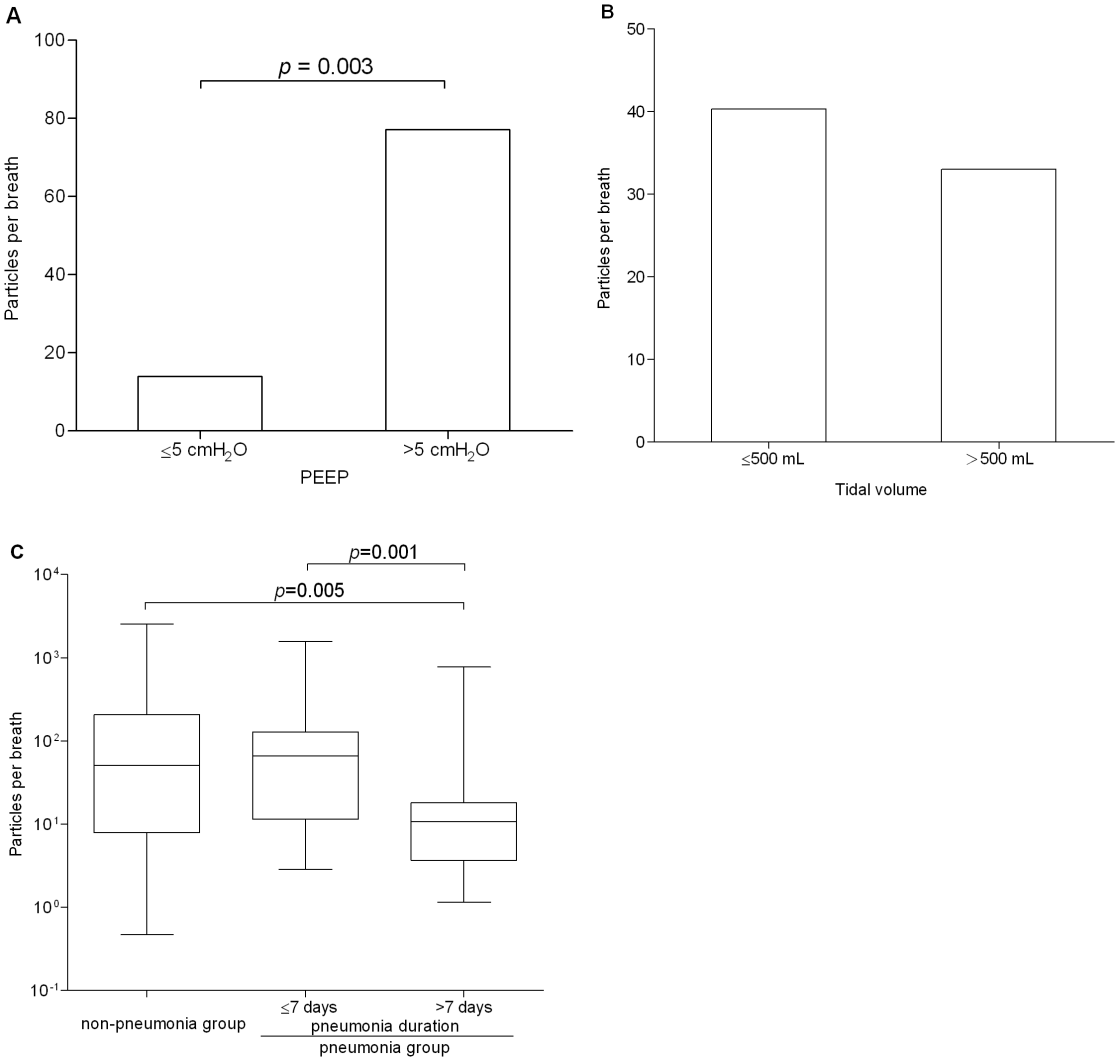
Association between EBP concentration and PEEP (A), tidal volume (B), and pneumonia (C).

In this study, the median EBP concentration of mechanically ventilated patients with the PC mode [33.88 particles/breath (0.062 particles/mL)] was similar to that for the VC mode [32.54 particles/breath (0.059 particles/mL), *p* = 0.455] (Fig. 5). To evaluate the relationships among EBPs concentrations, tidal volume and PEEP level, this investigation indicated that the median EBPs concentration from the mechanically ventilated patients with high PEEP level [77.08 particles/breath (0.184 particles/mL)] significantly exceeded that for patients with low PEEP level [13.92 particles/breath (0.027 particles/mL), *p* = 0.003] (Fig. 6A). However, the EBPs concentration was not associated with tidal volume in the mechanically ventilated patients (*p* = 0.923, Fig. 6B). Additionally, the median (25–75 percentiles) EBPs concentrations in the patients without pneumonia [50.20 particles/breath (0.1 particles/mL), 6.72–198.73 particles/breath (0.015–0.464 particles/mL)] resembled those in patients with short duration pneumonia (≤7 days) [65.57 particles/breath (0.143 particles/mL), 11.40–128.02 particles/breath (0.020–0.257 particles/mL), *p* = 0.628], but EBP concentrations clearly differed between the patients without pneumonia and those with long duration (>7 days) pneumonia [10.75 particles/breath (0.024 particles/mL), 3.67–18.04 particles/breath (0.008–0.039 particles/mL), *p* = 0.005]. The mechanically ventilated patients with short duration pneumonia displayed higher median EBP concentration than those with long duration pneumonia (*p* = 0.001) (Fig. 6C).

## Discussion

Until recently, few investigations evaluated EBPs size concentration distribution during normal breathing and coughing in mechanically ventilated patients with different ventilator settings. The inspiratory breathing circuit of the ventilator system was fitted with a HEPA filter to avoid the contamination of exhaled breath samples with external particles during the study period.

In the study, the EBP concentration and variation of the mechanically ventilated patients with normal breathing resembled those of the healthy adults (0.01– 2.1 particles/mL) in previous studies [1], [5], [6]. However, the EBPs concentration of mechanically ventilated patients might be underestimated, because these patients used 35 cm of artificial endotracheal tube with dead volume for EBPs deposition on the walls of endotracheal tubes.

Two mechanically ventilated patients with PC mode had high EBPs concentrations [1,037.27–1,163.87 particles/breath (1.415–2.527 particles/mL)]. One patient was diagnosed with rectal cancer, and his lung showed severe pulmonary edema and *Candida* infection. Another patient was diagnosed lung cancer and pneumonia, and *Acinetobacter baumannii* was found in the sputum of this patient. Furthermore, three mechanically ventilated patients with VC mode had higher EBPs concentrations [1,037.70–2,554.04 particles/breath (2.075–4.644 particles/mL)] than other mechanically ventilated patients. The possible reason for the production of high EBPs from the mechanically ventilated patients may relate to the patient diagnosis (pulmonary tuberculosis, lung cancer, and pneumonia). Increasing the sample sizes is suggested to evaluate the relationship between pulmonary disease and EBPs concentration.

Human actions such as coughing, sneezing, talking, singing, and breathing produce EBPs. This study reported that mechanically ventilated patients produced high EBPs concentrations during coughing. Similar results have previously been found in studies in England [11] and the USA [6]. However, the sample size of patients with coughing during mechanical ventilation is too small, so that the EBP concentration distribution of mechanically ventilated patients during coughing will deserve further evaluation. Furthermore, this investigation showed that the EBPs concentrations from the mechanically ventilated patients with the PC mode were similar to those of mechanically ventilated patients with the VC mode. This phenomenon possibly occurred because the recruited patieints were stable and so their respiratory parameters (tidal volume, oxygen demand, and respiratory rate) did not differ significnatly between ventilation modes. Moreover, the EBP size distributions in the mechanically ventilated patients with the PC mode resembled those for mechanically ventilated patients with the VC mode. All EBPs were sized below 5 µm and 80% of EBPs were sized between 0.3 µm and 1.0 µm. This result resembled those of previous studies involving healthy adults [1], [5], [6], [16] and patients with the common cold [17] that exhaled particles sized below 1 µm.

Previous investigations indicated that tidal volume and respiratory rate clearly increased EBPs concentrations, while inspiratory flow did not [2], [18]. High tidal volume increased the opportunity for the re-opening of terminal airways, so EBPs concentrations tended to be high in healthy adults. The current study showed that tidal volume was not associated with EBPs concentrations in mechanically ventilated patients. This result differed from that of previous studies [2], [18]. The possible reason for this phenomenon relates to the setting of tidal volume (10 ml/kg) based on personal ideal body weight. The mechanism of EBPs production in mechanically ventilated patients thus warrants further evaluation.

The setting of PEEP was used to open the alveolars of mechanically ventilated patients to increase gas diffusion, functional residual capacity and alveolar compliance, and then to improve oxygenation, and to decrease oxygen demand and work of breathing. Therefore, it was speculated that EBPs concentrations were lower when mechanically ventilated patients have high PEEP levels. However, this investigation showed that EBPs concnetrations from mechanically ventilated patients with high PEEP level exceeded those of such patients with low PEEP level. This result has two possible causes: 1) 60% of mechanically ventilated patients with high PEEP level had pneumonia; 2) 40% of mechanically ventilated patients with high PEEP level had severe pulmonary edema in X-ray images, and these patients were infected with *Candida*, *Acinetobacter baumannii*, and *Mycobacterium tuberculosis*. The relationships among pulmonary infiltration, PEEP level, and EBPs concentrations thus warrants further investigation.

Additionally, this study demonstrated that patients with pneumonia for over 7 days had much lower EBPs concentrations than those with pneumonia for less than 7 days. The reason for this difference may relate to a good clinical cure effect achieved through a week-long course of antibiotics in patients with penumonia. This phenomenon also suggests that EBPs concentration distributions should be evaluated before and after antibiotics therapy in mechanically ventilated patients with pneumonia. Furthermore, six mechanically ventilated patients had pulmonary infiltration and edema but without pneumonia (the sputum cultures identified *Mycobacterium tuberculosis* in two of the six patients) shown in chest X-ray image, and the EBPs concentrations of these patients [237.67– 1,208.13 particles/breath (0.2–2.196 particles/mL)] were higher than those of other patients without pneumonia [2.35–887.69 particles/breath (0.005–1.928 particles/mL)]. Since pathogens might exit the airways together with EBPs, it is necessary to evaluate the concentration distribution of exhaled breath bacteria (EBB) from mechanically ventilated patients. Furthermore, the large bacterial filters in the expiratory breathing circuits of ventilator systems, particularly those with repeated disinfection using an autoclave system, warrant further evaluation of their efficiency in the filtration of EBPs.

In conclusion, most EBPs in the mechanically ventilated patients were between 0.3 µm and 1.0 µm. The EBPs concentrations from mechanically ventilated patients were positively associated with PEEP level and negatively associated with pneumonia duration. This study found no relationship between EBPs concentrations and tidal volume.
